# A rare case of diffuse large B-cell lymphoma masquerading as Guillain Barre syndrome, Warburg phenomenon and hemophagocytic lymphohistiocytosis

**DOI:** 10.3332/ecancer.2025.2012

**Published:** 2025-10-09

**Authors:** Anupama Kurup, Deepak Charles

**Affiliations:** 1 Department of Internal Medicine, Aster Medcity, Kochi, Kerala 682027, India; 2 Haematology and Haemato-Oncology, Aster Medicity, Kochi 682027, Kerala, India; a https://orcid.org/0009-0006-7703-1114

**Keywords:** diffuse large B-cell lymphoma, Guillain–Barre syndrome, Warburg phenomenon, hemophagocytic lymphohistiocytosis

## Abstract

We report a case of pyrexia of unknown origin presenting with lactic acidosis and hypoglycaemia. He further developed Guillain–Barre syndrome (GBS) and Hemophagocytic lymphohistiocytosis (HLH). The bone marrow biopsy ultimately reported Diffuse large B cell lymphoma (DLBCL). A 74-year-old gentleman visited the hospital in January 2024 with a fever since 2 weeks. He further developed hypoglycemic episodes and lower limb followed by upper limb weakness. On physical examination, he was febrile, tachypnoec with hepatomegaly and bilateral lower limb weakness with grade 3 power proximally and extensor plantar reflexes. Peripheral smear showed a leucoerythroblastic picture and 3% atypical lymphoid cells. Procalcitonin, lactate dehydrogenase and Ferritin were markedly elevated, suggestive of HLH. The bone marrow biopsy ultimately revealed DLBCL. He was diagnosed with GBS, Warburg phenomenon and HLH secondary to DLBCL. Intravenous steroids were started for secondary HLH and after he became hemodynamically stable was initiated on chemotherapy with Rituximab, Cyclophosphamide, Doxorubicin, Vincristine, Prednisolone (R-CHOP) regimen. After four cycles of R mini-CHOP, positron emission tomography–computed tomography showed a good response. Further two cycles of R mini-CHOP was given with methotrexate for central nervous system prophylaxis. This case portrays an atypical presentation of DLBCL with Warburg syndrome, GBS and HLH. Despite the adverse clinical course, the patient responded favourably to chemotherapy.

## Background

The phenomenon of the Warburg effect was described in literature by Otto Warburg in 1920s. It is characterised by increased glucose uptake leading to extreme lactate production by tumour cells in the presence of oxygen [1]. It is a rare complication of non-tissue perfusion abnormalities caused by solid tumours or hematologic malignancies. Only a few cases of the Warburg effect with lactic acidosis and hypoglycaemia in lymphoma have been reported and are associated with a very grim prognosis, leading to 70%–80% mortality in the first month following diagnosis [2]. The definitive treatment for malignancy-associated lactic acidosis is chemotherapy.

Here we report a case of pyrexia of unknown origin (PUO) that presented with severe lactic acidosis and hypoglycaemia that ultimately turned out to be diffuse large B cell lymphoma (DLBCL). He further developed paraneoplastic Guillain–Barre syndrome (GBS) and Secondary Hemophagocytic lymphohistiocytosis (HLH) during the course of hospital stay. Even though his journey was quite complicated, it is one of the success stories of modern medicine.

## Case report

A 74-year-old gentleman, with no known comorbidities, visited the outpatient department of a local hospital in January 2024 with fever since 2 weeks. He had no other localising focus of infection. Routine investigations revealed elevated inflammatory markers (C-reactive protein-160 mg/L, ferritin −460 ng/mL and Lactate dehydrogenase (LDH) −928 IU/L). He was started on empirical antibiotics and other supportive measures. Peripheral smear revealed leukopenia with shift to left and thrombocytopenia of 70,000/u. Tropical fever panel work up turned out to be negative and he was evaluated as a case of PUO. All cultures were sterile and serum procalcitonin was negative. Rheumatological work up yielded no positive results. Arterial blood gas analysis revealed high anion gap metabolic acidosis with elevated lactate (11 mEq/L) and he started developing hypoglycaemic episodes. He further developed lower limb followed by upper limb weakness. Nerve conduction velocity revealed severe axonal sensory-motor neuropathy. Autoimmune panel was reported negative and lumbar puncture was acellular. He was treated with IV immunoglobulins and steroids, after which there was significant neurological improvement.

He was transferred to our centre for evaluation of PUO, bicytopenia, lactic acidosis and recurrent hypoglycemic episodes. On admission, he was tachypnoeic, tachycardic and febrile with mild hepatomegaly. He further developed hypotension and oliguria. Ionotropic supports and haemodialysis were initiated. Peripheral smear was repeated that showed leucoerythroblastic picture and 3% atypical lymphoid cells. Procalcitonin was markedly elevated along with high LDH (1,998 IU/L), ferritin (8,476 ng/mL) and triglyceride values (266 mg/dL) suggestive of HLH. Positron emission tomography–computed tomography (PET CT) showed persistent diffuse significantly increased FDG avidity in enlarged liver and spleen and persistent significantly increased fluorodeoxyglucose (FDG) avidity in marrow spaces of almost entire axial and proximal appendicular skeleton − suggestive of an infiltrative disease aetiology, likely hematogenous malignancy. Bone marrow aspirate report revealed a cellular marrow with megaloblastic erythroid hyperplasia with a mild increase in histiocytes and a few showing hemophagocytosis. Bone marrow biopsy was reported as cellular marrow with normoblastic erythroid hyperplasia and a cluster of large atypical mononuclear cells. The immuno histochemistry showed CD 20 positive, CD 10 negative, CD 34 negative, CD 117 negative, Kappa and lambda negative, consistent with high-grade B-cell lymphoma. Karyotyping analysis revealed t(1:1)(p36:q21) seen in lymphomas. Cerebrospinal fluid analysis was found to be normal.

Intravenous steroids were started for secondary HLH with tumour lysis syndrome prophylaxis. He was gradually tapered off Noradrenaline supports and procalcitonin showed a reducing trend. He became hemodynamically stable and was initiated on chemotherapy with Rituximab, Cyclophosphamide, Doxorubicin, Vincristine, Prednisolone (R-CHOP) regimen.

Post chemotherapy, he was complaining of urinary retention and faecal incontinence. Nervous system examination revealed bilateral lower limb weakness with grade 3 power proximally and extensor plantar reflexes. Magnetic resonance imaging whole spine with contrast was done, which revealed mild diffuse enhancement along the walls of thecal sac and cauda equina nerve roots with a small enhancing nodular focus noted in the right para centra location within cauda equina nerve roots at L1 level suggestive of conus cauda syndrome. Alpha blockers were added for an underactive bladder. Physiotherapy sessions were initiated and he was discharged with stable hemodynamic parameters.

After completing four cycles of RCHOP regimen, a PETCT was done that showed good response. He had a good neurological response on assessment during the follow up visits. Further two more cycles of R mini-CHOP were continued and is currently on high-dose methotrexate regimen for central nervous system prophylaxis.

## Discussion

DLBCL is the most common lymphoma, accounting for about 25% to 30% of all the non-Hodgkin lymphomas [3]. It usually presents as a rapidly growing mass or enlarging lymph nodes in a nodal or extranodal site. Even though it is an aggressive tumour, it responds well to six cycles of rituximab along with cyclophosphamide, doxorubicin, vincristine and prednisone (R-CHOP).

Paraneoplastic neurological syndromes are rarely associated with NHL, although it can be life-threatening at presentation. It is caused by immune mechanisms triggered against antigens that are normally present in the nervous system and ectopically expressed by the tumour. A literature review indicated that DLBCL patients with GBS were typically elderly; more than 80% of patients were male and aged over 60 years as our patient [4]. GBS can occur either prior to diagnosis or after diagnosis and treatment of lymphoma. The former, as in our case, is caused by tumour factors and the latter can be related to infection and neurotoxicity caused by chemotherapeutic agents. In these cases, glucocorticoids alone or in combination with plasmapheresis were used in the immunoglobulin pulse therapy for GBS. Chemotherapy protocols for CHOP±R and R-DA-EPOCH were chosen if the definitive diagnosis of lymphoma is made. Studies showed poorer outcomes in patients who developed GBS before lymphoma diagnosis and used only immunoglobulin pulse therapy [5]. Nevertheless, our patient had a good outcome with the use of IV immunoglobulins and steroids alone.

Another unique aspect of our case was the presence of recurrent hypoglycaemia without any underlying endocrinological disorder and persistent lactic acidosis. Here, the presence of lactic acidosis could not be explained by low perfusion status, toxins, drugs or thiamine deficiency and it persisted despite fluid replenishment and correction of acidosis [6]. The association between hypoglycaemia and lactic acidosis is a rare but well-documented occurrence in malignancies but its association with severe asymptomatic hypoglycaemia is an extremely rare phenomenon [7, 8]. Type B lactic acidosis is a life-threatening disease that mainly occurs in patients with haematological malignancies but can also occur with solid tumours [9]. Elhomsy *et al* [10] showed in their literature that aggressive glucose infusions to correct hypoglycaemia would not elevate glucose levels but increase lactate production by feeding the tumour cells and promoting aerobic glycolysis.

In our case, even though the possibility of an underlying malignancy (mostly haematological) was considered from outside the hospital, we were not able to reach a conclusion on the blood lineage, as immunohistochemistry markers were modified by steroid administration. Type B lactic acidosis associated with malignancy carries a poor prognosis with a mortality rate of around 90% [11]. The best treatment for the Warburg effect in haematological malignancy is not yet known. But the commonly used modalities for treatment include chemotherapy, bicarbonate infusion, renal replacement therapy and insulin infusion, all of which have been used in our case [12]. Amongst all, initiating aggressive chemotherapy has been the most effective in the reversal of acidosis, as seen in our case as well.

To further add on to the list of complications, our patient developed HLH. Our patients’ findings are suitable for six out of eight criteria of hemaphagocytic syndrome, which are as follows: fever 38.5ºC, bicytopenia, hemophagocytosis in bone marrow, hypertriglyceridemia, reduced fibrinogen and increased ferritin. HLH is a life-threatening clinical condition and should promptly be treated. It can also be triggered by a superadded infection in an underlying malignancy [13]. The mainstay treatment of malignancy-associated HLH is chemotherapy, which was promptly initiated in our case.

This case report portrays an atypical presentation of DLBCL in the context of Warburg syndrome with quite a few complications heralding the malignancy − namely GBS, HLH and cord compression. Despite the adverse clinical course, the patient responded favourably to chemotherapy and is at present finished six cycles of chemotherapy and doing well.

## Conflicts of interest

The authors declared no potential conflicts of interest with respect to the research, authorship and/or publication of this article.

## Funding

The authors received no financial support for the research, authorship and/or publication of this article.

## Informed consent

Informed consent to publish this case was obtained from the patient included in this case report.

## Author contributions

Anupama Kurup: conceptualised the case report, reviewed the literature and drafted the manuscript.

Deepak Charles: Analysed patient data, performed the clinical management and revised the manuscript critically and proof readed for accuracy.
